# Dual-ligated metal organic framework as novel multifunctional nanovehicle for targeted drug delivery for hepatic cancer treatment

**DOI:** 10.1038/s41598-021-99407-5

**Published:** 2021-10-06

**Authors:** Mostafa Fytory, Kholoud K. Arafa, Waleed M. A. El Rouby, Ahmed A. Farghali, Mahmoud Abdel-Hafiez, Ibrahim M. El-Sherbiny

**Affiliations:** 1 Nanomedicine Labs, Center for Materials Science (CMS), Zewail City of Science and Technology, Giza, 12578 Egypt; 2 Material Science and Nanotechnology Department, Faculty of Postgraduate Studies for Advanced Sciences (PSAS), Beni-Suef University, Beni-Suef, 62511 Egypt; 3 Department of Physics and Astronomy, Uppsala University, Box 516, 75120 Uppsala, Sweden; 4 Department of Physics, Faculty of Science, Fayoum University, Fayoum, 63514 Egypt

**Keywords:** Nanomedicine, Nanoparticles

## Abstract

In the last decade, nanosized metal organic frameworks (NMOFs) have gained an increasing applicability as multifunctional nanocarriers for drug delivery in cancer therapy. However, only a limited number of platforms have been reported that can serve as an effective targeted drug delivery system (DDSs). Herein, we report rational design and construction of doxorubicin (DOX)-loaded nanoscale Zr (IV)-based NMOF (NH_2_-UiO-66) decorated with active tumor targeting moieties; folic acid (FA), lactobionic acid (LA), glycyrrhetinic acid (GA), and dual ligands of LA and GA, as efficient multifunctional DDSs for hepatocellular carcinoma (HCC) therapy. The success of modification was exhaustively validated by various structural, thermal and microscopic techniques. Biocompatibility studies indicated the safety of pristine NH_2_-UiO-66 against HSF cells whereas DOX-loaded dual-ligated NMOF was found to possess superior cytotoxicity against HepG2 cells which was further confirmed by flow cytometry. Moreover, fluorescence microscopy was used for monitoring cellular uptake in comparison to the non-ligated and mono-ligated NMOF. Additionally, the newly developed dual-ligated NMOF depicted a pH-responsiveness towards the DOX release. These findings open new avenues in designing various NMOF-based DDSs that actively target hepatic cancer to achieve precise therapy.

## Introduction

Metal Organic Frameworks (MOFs) are a promptly growing class of porous material with extraordinary features wherefore were emerged in various applications such as catalysis^1^, sensing^2^, optics^3^, gas adsorption^4^, and drug delivery^5,6^. Nanosized MOFs (NMOFs) exhibit unique characteristics such as being highly ordered structures with a large surface area and a large pore volume, that could enhance their ability to entrap the active molecules through saturation of these molecules on their surface and into their highly porous structure^7^. Furthermore, active molecules could be incorporated into NMOFs structure through covalent bonds via one-pot synthesis or a post-synthesis modification. These remarkable characteristics together with biocompatibility, low toxicity, and high loading capacity nominated NMOFs as potential platforms for various biological applications, especially as smart drug delivery vehicle for cancer treatment^6,8,9^.

Hepatocellular carcinoma (HCC) is one of the most critical diseases affecting human health, and its morbidity has steadily increased. While cancer therapies have been improved and the survival rates have recently increased, effective cancer treatments remain a massive challenge for physicians and researchers globally^10^. Several approaches were applied to HCC treatment including surgery, radiation, immunotherapy, and chemotherapy^11^. However, inadequate biodistribution, high drug doses, and lack of targeting ability to cancer cells remains the main drawbacks for the chemotherapy strategies^12^. Accordingly, this necessitates the creation of innovative and effective drug delivery systems (DDSs). Recently, NMOF nanocarriers have been shown to achieve targeted drug delivery, increased cellular absorption, and managed drug release, delineating them as a promising class of DDSs, incorporating chemotherapeutic agents^13,14^. Chemotherapeutic agents can be selectively targeted to tumors using different methods, including passive targeting, active targeting, and through using stimuli-responsive systems. In the active targeting, the DDSs deliver the bioactive cargo to the targeted part due to the high affinity between ligands linked to the DDS and the receptors that are overexpressed on the surface of the targeted tumor cell^15^. Numerous targeting ligands have been identified to target the hepatic tumor cells including antibodies, polysaccharides, peptides, and aptamers. Examples for such ligands are folic acid (FA), lactobionic acid (LA), glycyrrhetinic acid (GA), hyaluronic acid (HA), galactose, and biotin^16^. Furthermore, some studies reported the use of multiple ligands for active targeting in order to increase the efficiency and selectivity of the targeting therapy system^17,18^.

To enhance the targeting characteristics of NMOFs, several targeting moieties have been applied individually to NMOFs such as FA and HA^19–22^. However, it was hypothesized that adding more than one targeting ligand to NMOF surface could enhance its delivery and targeting performance to cancer cells.

The current study aims at reporting the development, physiochemical characterization, and biological evaluation of doxorubicin (DOX)-loaded NH_2_-UiO-66 NOMF and functionalized with either mono-targeting or dual-targeting moieties including FA, GA and LA for the selective liver carcinoma targeting. Based on the optimal monoligated-NMOF results, the ligands; GA and LA were used to develop a dual-ligated NH_2_-UiO-66 NOMF DDS. Worthy to note that the approach of using a dual-targeting NMOF DDS to HCC was not reported in the literature, and this study is the first report in this regard. The obtained results showed a high efficiency in both cellular uptake and cytotoxicity behavior of the developed dual-ligated NMOF as compared to either non-ligated or the various developed mono-ligated NMOF.

## Materials and methods

### Materials

2-Aminoterephthalic acid (NH_2_-BDC 95.5%), zirconium tetrachloride (ZrCl_4_, 99.5%), folic acid (FA), *N*-(3-dimethylaminopropyl)-*N*′-ethyl carbodiimide hydrochloride (EDC.HCl), and N-hydroxy succinimide (NHS) were obtained from Sigma-Aldrich (Darmstadt, Germany). 8β-glycyrrhetinic acid (GA) and lactobionic acid (LA) were purchased from Acros Organics (New Jersey, USA). Doxorubicin hydrochloride (DOX.HCl), hydrochloric acid (HCl), glacial acetic acid (CH3COOH), and anhydrous *N*,*N*-dimethyl formamide (DMF) were all purchased from Sigma-Aldrich (St Louis, MO, USA). Fetal bovine serum (FBS), Dulbecco’s Modified Eagle Medium (DMEM), penicillin–streptomycin and trypsin–EDTA (0.25%) were purchased from Gibco. All other reagents and solvents were of analytical grade and were used as received without further purification.

### Synthesis of nanosized NH_2_-UiO-66 NMOF

Nanosized NH_2_-UiO-66 (NMOF) was synthesized as reported elsewhere^23^ with slight modifications. Briefly, ZrCl_4_ (0.5 g, 2.14 mmol) was injected into a vial containing a mixture of 15 ml DMF and 4 ml HCl before sonication for 20 min. Afterwards, the solution (0.536 g, 2.9 mol) of NH_2_-BDC in 15 ml DMF was added and sonicated for an additional 20 min before heating for 24 h at 80 °C. Finally, the solution was cooled with air, and the product was collected. The precipitate was soaked in 10 ml DMF and washed several times with acetonitrile. The precipitate was then dried under vacuum at 60 °C overnight. The obtained NMOF was activated at 150 °C for 4 h before use.

### Synthesis of targeting ligands modified NH_2_-UiO-66 NMOF

Three liver targeting moieties (FA, LA, and GA) were chosen in this study to be conjugated to the developed nanosized NH_2_-UiO-66 (NMOF) to design novel DDSs. Briefly, the carboxylic groups of the targeting ligands were activated through EDC.NHS chemistry. Three different molar ratios (1:8, 1:4 and 1:2) between the pristine NMOF and the activated carboxylic groups of the targeting ligands were applied and reacted in 30 ml of DMF under vigorous stirring for 72 h, as described in Table S1 (supplementary information). Then, the mixtures were pelleted by centrifugation (9000 rpm, 20 min × 2), and washed twice with DMF/water (v/v, 1/99) to remove excessive targeting ligands. Finally, the obtained ligated-NMOF were immersed in 30 ml of n-hexane for 4 h for solvent exchange, followed by vacuum drying at 100 °C for 12 h.

### Preparation of DOX-loaded NH_2_-UiO-66 NMOF and determination of the DOX entrapment efficiency (EE%) and loading capacity (LC%)

The physical DOX loading to all NMOFs formulations was carried out in a sealed vessel at dark condition with stirring for 72 h. Simply, 100 mg from each formula was mixed with 35 mg of DOX in an aqueous media. Then, pelleting by centrifugation and washing several times with water were conducted before vacuum drying at 40 °C. Eventually, the supernatants were analyzed to detect the unentrapped DOX. The DOX entrapment efficiency (EE%) was calculated using the following equation:

$${\text{Entrapment}}\, {\text{efficiency}}\, {\text{EE}}\% = \frac{{{\text{Drug}}_{{total}} - {\text{Drug}}_{{unloaded}} }}{{{\text{Drug}}_{{total}} }} \times 100$$

where Drug _*Total*_ is the original weight of drug (DOX.HCl) before carrying out the experiment whereas Drug _*unloaded*_ is the free drug weight in the centrifugal aliquot.

The loading capacity (LC%) was calculated using the following equation:

$${\text{LC }}(\% ) = \frac{{({\text{Drug}})_{{total}} {\text{ }} - ({\text{Drug}})_{{unloaded}} }}{{({\text{Drug}} + {\text{NMOF}})_{{total}} }} \times 100$$

where, (NMOF)_*total*_ is the total weight of NMOF initially added to the aqueous solution.

### Physicochemical characterization of the developed plain and DOX-loaded NMOFs

Fourier transform infrared spectra (FTIR) in the range 400–4000 cm^−1^ were collected as KBr pellets using a VERTEX 70 FTIR spectrophotometer 16 (Bruker Optics, Germany). X-ray powder diffraction (PXRD) of the synthesized formulations were performed using (PANalytical Empyrean, Switzerland) operating with (monochromated Cu-Kα2, k = 1.548 Å; 40.0 kV, 35.0 mA). Field emission scanning electron microscopy (FE-SEM) images were acquired exploiting a Quanta FEG 250, Switzerland for studying the morphology of the prepared formulations. The size and shape of selected NMOFs was examined using transmission electron microscopy (TEM; CEM 902A; Carl Zeiss, Oberkochen, Germany). Thermogravimetric analyses (TGA) measurements were carried out via heating samples at a rate of 10 °C min^−1^ in nitrogen gas from ambient temperature to 700 °C. Differential scanning calorimetry (DSC) thermograms were generated using (Mettler Toledo, Changzhou, China) DSC system. The NMOF materials were heated from 20 to 400 °C at 10 °C min^−1^ rate. The Brunauer–Emmett–Teller (BET) surface area for the prepared NMOFs formulations was determined from the nitrogen adsorption/desorption isotherms and was performed by (Micromeritics Tri Star II). Surface zeta potential of the different developed plain and ligated NMOFs were performed at 25 °C using electrophoretic light scattering (ELS) methods with the aid of Malvern zetasizer. UV–vis is spectrophotometer (Thermo Scientific Evolution 600, USA) was employedF to measure both EE% and LC%, and the drug release profile from the NMOFs carriers.

### Drug release experiment

Drug release from DOX-loaded LA-GA dual-ligated NMOF into phosphate buffer solutions, PBS (pH 7.4 and 4) was measured by a dialysis technique. A dialysis bag (molecular weight cut off 1200 KDa) containing 10 ml suspension of DOX-loaded LA-GA-NMOF was incubated in the PBS (pH 4 or 7.4) at 37 °C ± 1 °C under gentle stirring (110 rpm). At certain time intervals during the dialysis process, an aliquot of 1 mL was taken from the supernatant of the PBS then replaced with the same volume of fresh PBS. The amount of the DOX in the collected supernatant aliquots was monitored by UV–vis is at 480 nm.

### Biocompatibility assay of NMOF on human fibroblast skin (HFS)

Cell viability was tested by the SRB assay. Aliquots of 100 μl cell suspension (5 × 10^3^ cells) were placed in 96-well plates and incubated in complete media for 24 h. Cells were treated with another aliquot of 100 μl media containing drugs at various concentrations ranging from (10, 50, 100, 500, and 1000 μg/ml). After 72 h of drug treatment, cells were fixed by replacing media with 150 μl of 10% TCA and incubated at 4 °C for one hour. The TCA solution was removed, and the cells were washed 5 times with distilled water. Aliquots of 70 μl SRB solution (0.4% w/v) were added and incubated in a dark place at room temperature for 10 min. Plates were washed 3 times with 1% acetic acid and allowed to air-dry overnight. Afterwards, 150 μl of TRIS (10 mM) was added to dissolve protein-bound SRB stain, and the absorbance was measured at 540 nm using a BMG LABTECH®-FLUOstar Omega microplate reader (Ortenberg, Germany).

### In-vitro cytotoxicity study

Hepatocellular carcinoma (HepG2) cells were treated by the developed six formulations, NMOF, FA-NMOF, LA-NMOF, GA-NMOF and LA-GA-NMOF. Cells were cultured using DMEM (Invitrogen/Life Technologies) supplemented with 10% FBS (Hyclone), 10 ug/ml of insulin (Gibco), and 1% penicillin–streptomycin (Gibco). All the other chemicals and reagents were from Gibco-Germany; Invitrogen, ThermoScientific, Germany. The cells were plated (cells density 1.2–1.8 × 10^3^ cells/well) in a volume of a 100 µl complete growth medium + 100 µl of the tested formula per well in a 96-well plate for 24 h before the treatment with tested formulations. The next day, the tested formulations were prepared at five dilutions (100, 10, 1, 0.1 and 0.01 µmol) for each compound for treatment of cells. Finally, the cultured cells were treated with the tested compounds in triplicates, incubated for 24 and 48 h at 37 °C and 5% CO_2_. The cell proliferation assay was performed using the Vybrant® MTT cell proliferation assay kit, cat no: M6494 (Thermo Fisher, Germany). For the adherent cells, the medium was removed and replaced with 100 µl of fresh culture medium. Ten µl of the 12 mM MTT stock solution was added to each well, a negative control of 10 µl of the MTT stock solution was added to 100 µl of medium alone. The cells were incubated at 37 °C for 4 h. Subsequently, 100 µl of the SDS-HCl solution was added to each well and mixed thoroughly using a pipette. After 24 h, the MTT solution was removed and 100 μl of dimethyl sulfoxide was added to the wells. Cell viability was determined by measuring the optical density at 570 nm on a spectrophotometer (ELx 800; Bio-Tek Instruments Inc., Winooski, VT, USA). The IC_50_ was calculated using the Graph pad prism software 8.4.2.

### Cellular uptake study by fluorescence microscopy

The cellular uptake was collected by LABOMED Fluorescence microscope LX400, cat no: 9126000; USA and images were collected using OptikaI Sveiw software. The images were collected with two filters, which include: Drug (470/595 nm) “emits red color” and DAPI (340/452) “emits blue color”. A fusion of red and DAPI emitted pink color. The same procedure was repeated after 24 h for a 2nd set of treated cells. Briefly, cells were incubated in a 25 cm^2^ flask, the RPMI-1640 culture media in (Gibco, Thermoscientific, Germany) containing 10% fetal bovine serum (FBS) (Gibco, Thermoscientific, Germany) and 1% of penicillin G sodium (10,000 UI), streptomycin (10 mg) and amphotericin B (25 μg) (PSA) (Gibco, Thermoscientific, Germany) was used for culture. Then, cells were incubated at 37 °C in an atmosphere of 5% CO_2_. The cells are cultured 24 h before cellular uptake assessment. Afterwards, the HepG2 cells were treated with all formulations. The dose of DOX in all formulations was adjusted to 8.5 µmol/ml. The cells were incubated for two different time intervals, 24 h and 48 h.

### Cell apoptosis analysis by flow cytometry

Cells were harvested at 48 h post-transfection. Following trypsinization, FITC-annexin V and propidium iodide (PI) staining were performed. The dead cell apoptosis kit with annexin V-FITC and PI, for flow cytometry (Invitrogen, cat no: V13242) was used to separate apoptotic cells (early). The monoclonal antibodies detect the externalization of phosphatidylserine in apoptotic cells using recombinant annexin V which is conjugated to green-fluorescent FITC dye and dead cells using PI, where the PI stains necrotic cells with red fluorescence. After the treatment with both probes, apoptotic cells show green fluorescence, dead cells show red and green fluorescence, and live cells show little or no fluorescence. The Navios software (Beckman Coulter) was used to analyze flow cytometry data.

### Statistical analysis

All experimental data were represented as mean ± SD. The statistical analysis was performed by a two-way ANOVA. The calculations were done using Origin Pro software 64-bit. Differences with *P* < 0.05 were considered statistically significant.

## Results and discussion

**Figure 1:**
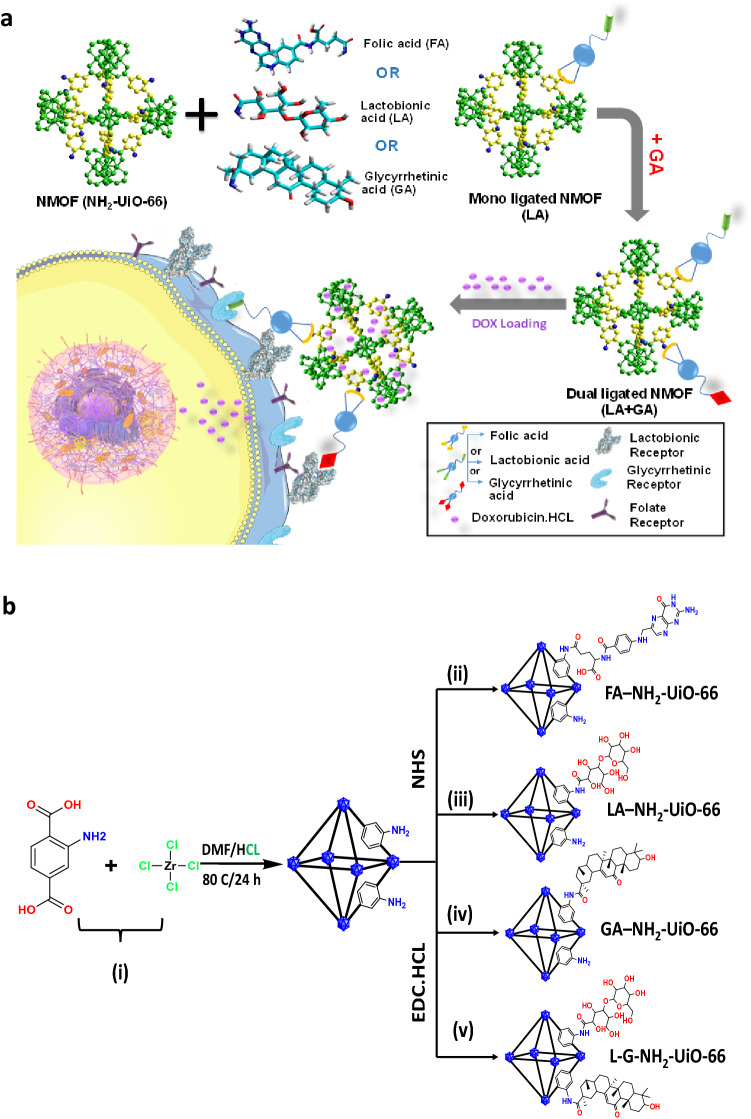
(**a**) Systematic representation of the functionalized Zr-based NMFOs (NH_2_-UiO-66) drug delivery carriers for targeted treatment of hepatocellular carcinoma. (**b**) The chemical synthesis scheme of mono- and dual ligated NH_2_-UiO-66. (**a**) was generated with HyperChem professional version 8.0.8 (http://www.hypercubeusa.com/), and (**b**) was generated with ChemDraw Professional version 18.1 (https://perkinelmerinformatics.com/).

NH_2_-UiO-66 NMOF could be a promising candidate of DDSs for effective multifunctional drug delivery because it exhibits several desirable characteristics. NH_2_-UiO-66 shows a high degree of connectivity in the crystal structure, providing outstanding chemical stability in a wide range of pHs in comparison with other MOFs-based drug delivery carriers^23–28^. Furthermore, the high surface area and the large internal pores size which facilitate entrapment of large chemotherapeutics agents such as DOX, as well as its high biocompatibility, low toxicity and the ability to be scaled down to nanoscale (100–200 nm) which is suitable for chemotherapy strategies^7^, and finally the presence of amino group facilitates the surface modification^28^ via covalent linking with small molecular targeting moieties (such as FA, LA, and GA), which are commonly used as targeting agents for HepG2 cells receptors^18,29^. The current study involves the design, synthesis, characterization and biological evaluation of biocompatible NH_2_-UiO-66 NMOF conjugated with either the mono-ligand (FA, LA or GA) or (LA and GA) as a dual-ligated NMOF, which preferentially localizes within the tumor mass. To our knowledge, this is the first attempt to develop a dual-functionalized Zr-based NMOF to achieve active targeting of the DOX.HCl at the cellular level as illustrated in Fig. 1a. The newly-developed dual-functionalized NMFO drug delivery carrier was assessed in-vitro for the HCC treatment.

### Synthesis of non-ligated, mono-ligated, and dual-ligated NH_2_-UiO-66 NMOFs

The synthesis strategy for the preparation of the UiO-66-NH_2_ NMOF and its post-modification with targeting moieties is shown in Fig. 1b. The UiO-66-NH_2_ NMOF was fabricated by heating a mixture solution of DMF and HCl containing ZrCl_4_ and 2-amino terephthalic acid ligand at 80 °C for 24 h. Post-synthetic modifications for the as-synthesized UiO-66-NH_2_ NMOFs were utilized to design a novel active targeting DDSs for HCC treatment. This was achieved via covalent linking to form amide bonds between the terminal carboxylic groups of targeting moieties (FA, LA, and GA), and the amine groups-containing NMOF with the aid of EDC.HCl/NHS as coupling agent to prepare either mono- or dual-ligated NMOF by click chemistry as illustrated in Fig. 1b.

### Characterization of the non-ligated and mono/dual-ligated NH_2_-UiO-66 NMOF

**Figure 2:**
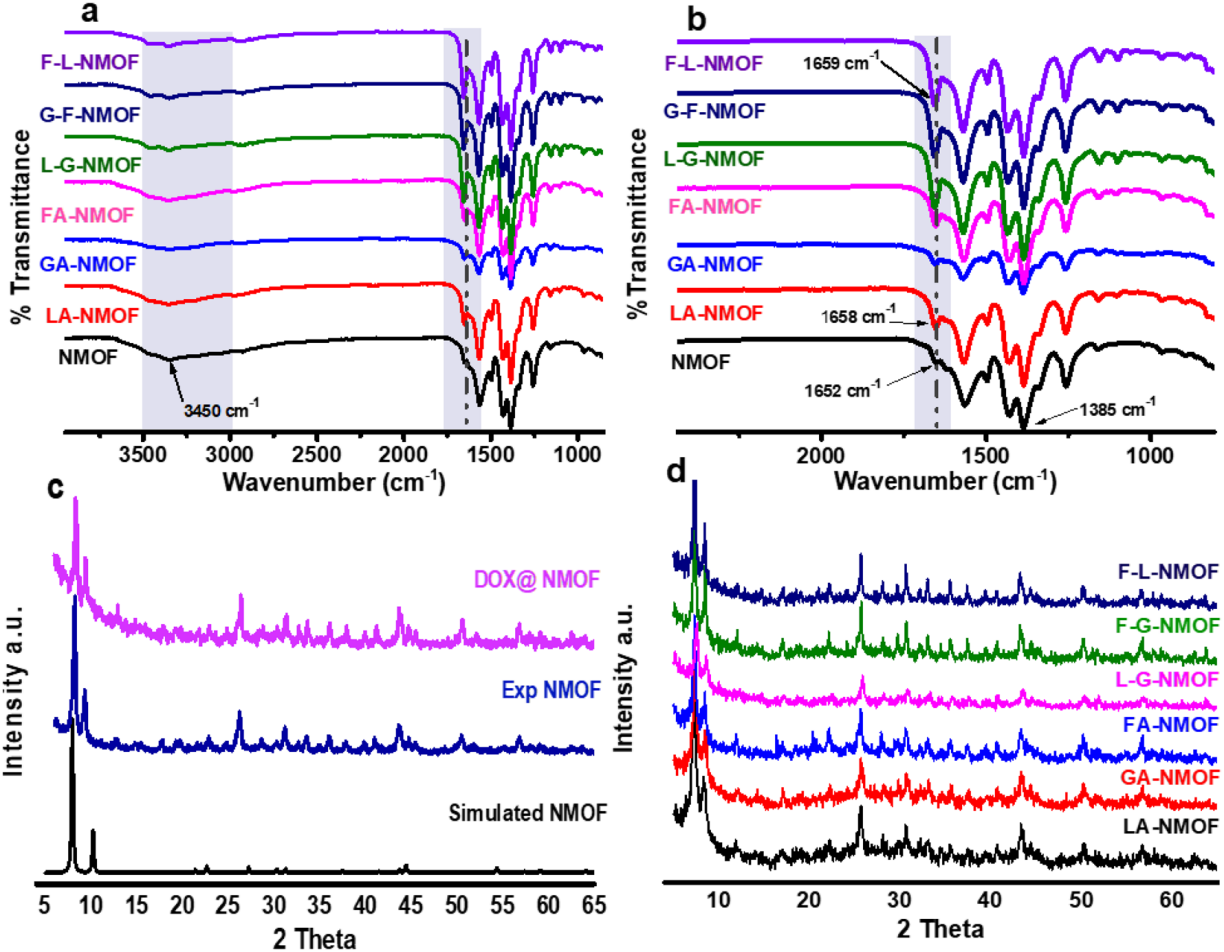
(**a**, **b**) FTIR spectra of NMOF, FA-NMOF, LA-NMOF, GA-NMOF, FA-LA-NMOF, GA-FA-NMOF and LA-GA-NMOF (**c**) PXRD for simulated NMOF, exp NMOF and DOX-loaded NMOF, and (**d**) PXRD for mono- and dual-ligated NMOF.

In order to understand the molecular structure of both the as-fabricated NMOF and ligated NMOF, FTIR measurements were performed. The spectra are shown in Fig. 2a,b. Both characteristic peaks due to symmetric and asymmetric vibrations were detected. The appearance of peaks at 1652 cm^−1^ and 1385 cm^−1^ were attributed to the asymmetric and symmetric vibration of the C=O and C=C groups, respectively. The peak at 3450 cm^−1^ was assigned to N–H bonds of amino terephthalic acid organic linker. In Fig. 2a and b the right grey shadow area shows a proof for conjugation between the amino group of the organic linker and the terminal carboxylic groups of targeting moieties via formation of amide bond that obviously appeared due to shifting the carbonyl group of NMOFs to red as well as increasing the intensity of the peak from mono-ligated NMOF to dual-ligated. In addition, the strong and broad absorption bands observed in the range of 3200–3600 cm^−1^ could be due to the presence of primary amines, the NH_2_ group as well as the 2‐aminoterephthalic acid in the NH_2_‐UiO‐66^30,31^. The NH_2_-UiO-66 shows a characteristic band at 3450 cm^−1^ that is assigned to the NH_2_ group and the decrease in the intensity of peak at 3450 cm^−1^ could be attributed to the consumption of the amine groups in conjugation with terminal carboxylic group of targeting moieties.

Furthermore, PXRD was used to study the crystallinity of the as-prepared NMOF, DOX-loaded NMOF, LA-NMOF, GA-NMOF, FA-NMOF, LA-GA-NMOF, FA-GA-NMOF, and FA-LA-NMOF. As shown in Fig. 2c, the PXRD patterns of NMOF and DOX-loaded NMOF are identical to the simulated pristine NMOF, hence confirming that the crystallinity and structural characteristics of the NH_2_-UiO-66 framework were maintained during DOX-loading. Furthermore, the DOX loading did not lead to additional diffraction peaks signifying the amorphous nature of the drug molecule entrapped inside the NMOF cavities. Thus, the physical encapsulation of the drug has no impact on the crystallinity of parent NMOF. Figure 2d indicated the post-modification PXRD pattern for both mono-ligated and dual-ligated NMOF. It can be also noted that in the case of LA which is of amorphous structure, the immobilization had no impact on the as-synthesized NMOF crystallinity. At the meantime, PXRD patterns of FA and GA ligated-NMOFs did not apparently change compared with their parent NMOFs, demonstrating that the crystallinity of UiO-66 was maintained even after post-immobilization of the targeting moieties to the surface of the as-fabricated NMOF.

### Thermal analysis (DSC and TGA) and surface area measurements

**Figure 3:**
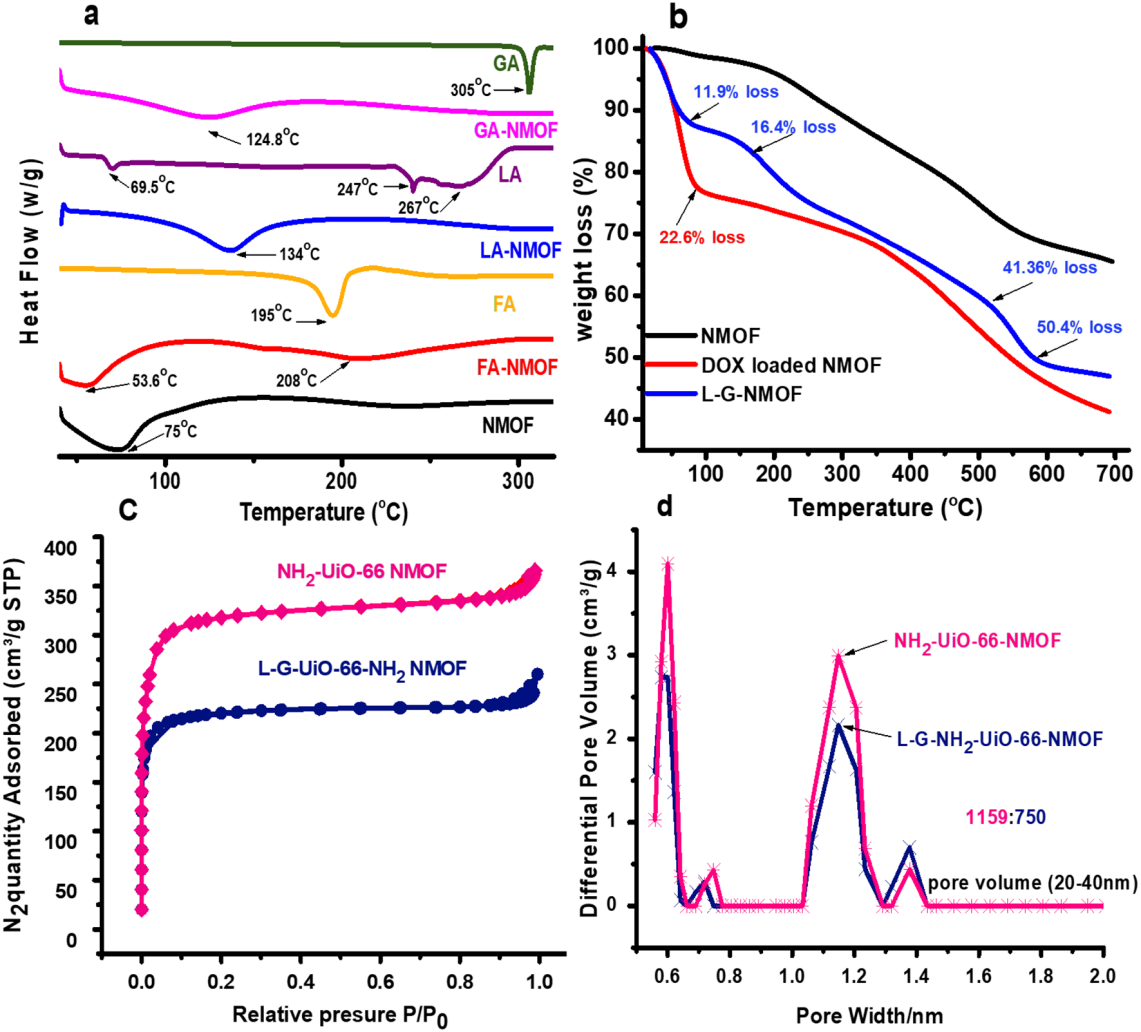
(**a**) DSC measurement for NMOF, mono-ligated NMOF, and the free FA, LA and GA ligand, (**b**) TGA analysis for NMOF, LA-GA-NMOF and DOX-loaded NMOF, (**c**) N_2_ adsorption and desorption isotherms at 77 K for NMOF and LA-GA-MOF, and (**d**) PSD for NMOF and LA-GA-NMOF.

The success of post-modification of targeting moieties and the thermal stability were further proven by DSC. As shown in Fig. 3a, the NMOF thermogram displayed an endothermic peak at 75 °C that could be assigned to the loss of H_2_O molecule bound to free NH_2_ Groups. Moreover, the thermogram showed NMOF thermal stability at elevated temperatures. As a proof for immobilization of targeting moieties on the surface of NMOF, it could be seen in Fig. 3a that the dehydration endothermic peak of FA-NMOF was shifted to 53.6 °C with a decrease in peak intensity which could be attributed to the consumption of amino groups in covalent linking between NMOF and FA. In the case of LA, as apparent in Fig. 3a, it exhibited small endothermic peaks at almost 69.5 °C, 240 °C and 267 °C. The thermogram of LA-NMOF showed the endothermic peak at 134 °C which could be assigned to dehydration of water molecules as well as drop of the intensity of dehydration endothermic peak at 75 °C, which proves the conjugation process between LA and NMOF. For the GA thermogram, it showed a sharp endothermic peak at 305 °C that is attributed to the GA decomposition, and for the GA-NMOF thermogram, it showed a small peak at 124.84 °C that corresponds to water molecules loss as well as the shift in the endothermic peak confirms the conjugation process.

For further confirmation of the success of conjugation and thermal stability, TGA measurements were performed under nitrogen atmosphere to NMOF, DOX-loaded NMOF, and LA-GA-NMOF as a representative of the dual-ligated NMOF. As shown in Fig. 3b, TGA measurement results indicate that NMOF could maintain its structural framework before 500 °C. The TGA curves of LA-GA-NMOF are in agreement with that reported in the literature, which indicated that LA has four weight loss steps at the temperature range from 30 to 600 °C^32^. As illustrated in Fig. 3b, LA-GA-NMOF showed the first mass loss with 11.9% between 30 and 130 °C, the second loss appear at temperature between (132–195 °C), with 16.4%, where the third and fourth weight losses occurred between (195–460 °C) and (470–600 °C), respectively with 41.36% and 50.4% mass loss. Furthermore, the increase in mass loss by 22.6% at 88 °C of DOX-loaded NMOF in comparison with pristine NMOF confirms the drug encapsulation as well. Moreover, N_2_ adsorption/desorption isotherm at 77 K as well as the pore size distribution of NMOF and LA-GA-NMOF are given in Fig. 3c and d. The calculated Brunauer–Emmett–Teller (BET) surface area values for NMOF demonstrated 1159 m^2^/g while the surface area of LA-GA-NMOF showed 750 m^2^/g. The observed decrease in the specific surface area of the LA-GA-NMOF compared to that of the plain NMOF can be ascribed to the success of conjugation of dual-ligands on the surface of NMOF.

Furthermore, the pore size distribution (PSD) histograms for the NMOF and LA-GA-NMOF are shown in Fig. 3d. The PSD of the LA-GA-NMOF closely resembles that of the NMOF, demonstrating two main pore systems centered at 0.6 and 1.2 nm. This assumption is further supported by the observed PSD for LA-GA-NMOF demonstrating major contribution by pores on the same width to that of the NMOF (20–40 nm).

### Surface morphology and topography of NMOFs formulations

**Figure 4:**
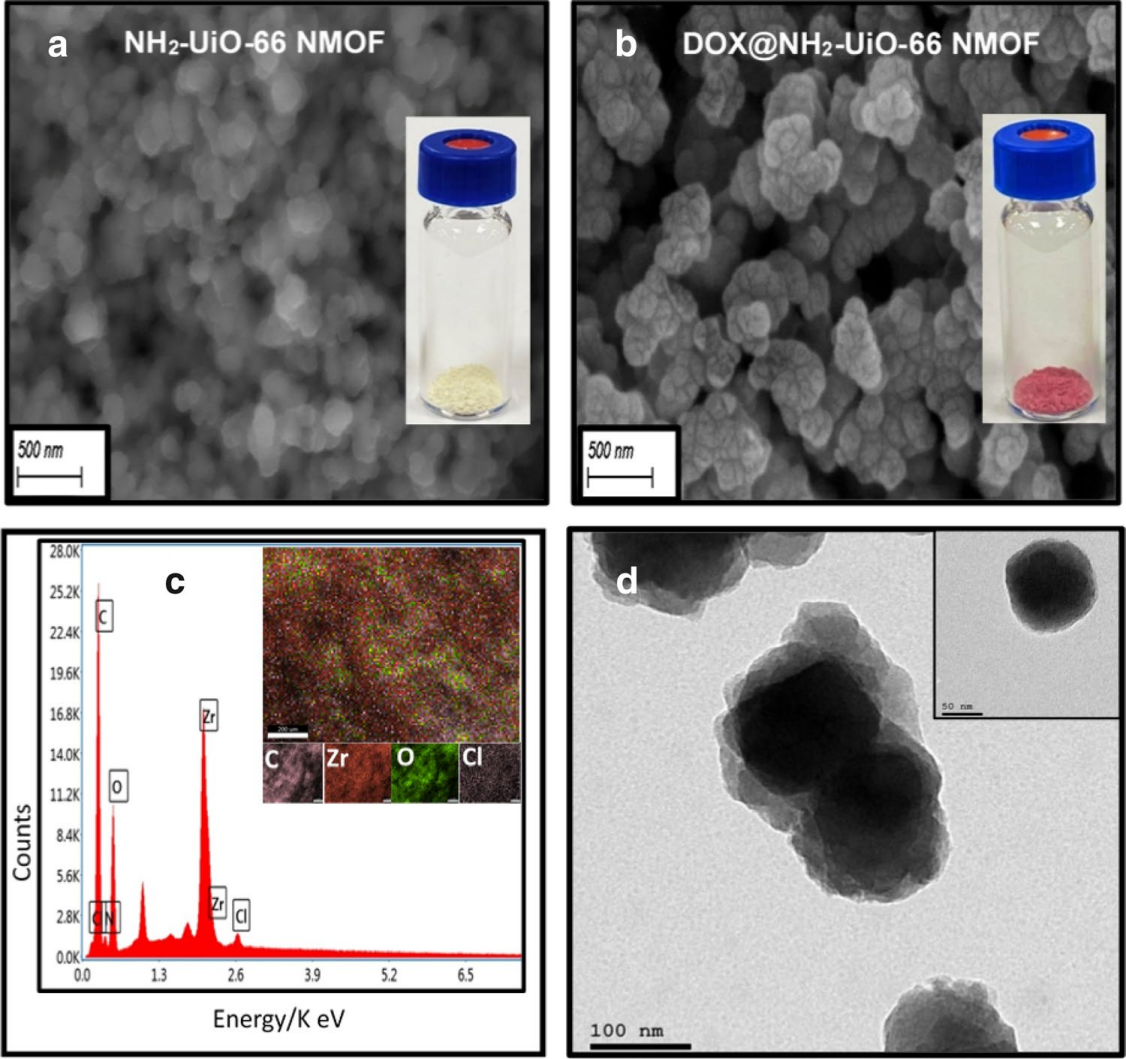
(**a**) SEM for plain NMOF (**b**) DOX-loaded NMOF and at upper right two images showing the physical appearance for both of NMOF and DOX-loaded NMOF, respectively, (**c**) Elemental mapping of DOX-loaded NMOF, (**d**) TEM of DOX-loaded NMOF, with a focus on single MOF crystal (inset).

SEM was applied to evaluate the structural morphology and particle size of the synthesized NMOF before and post DOX.HCl drug loading. As shown in Fig. 4a and b, it was found that the morphological characteristics of NMOF were maintained after DOX loading. The obtained images showed that most of the NMOF particles diameter range from 100 to 150 nm, which can allow their engulfment by cell receptor mediated endocytosis^33^. Consistently, the color of NMOF, has changed from yellow to red as shown in the upper right insets of Fig. 4a and b respectively, which confirmed the physical loading of red color DOX in the pores of the NMOF. Additionally, energy-dispersive X-ray spectroscopy (EDX) was used to verify the DOX loading into the porous NMOF support. In Fig. 4c, the consistency of NMOF was verified, where the Zr, O and C maps were noted, with the appearance of Cl element which could be attributed to DOX.HCl, and thus proving the physical loading of the drug. Furthermore, the TEM images for DOX-loaded NMOF showed a narrow-dispersed particles size distribution ranging from 90 to 120 nm as can be noted from Fig. 4d.

Loading of the DOX into the different NMOFs was carried out via incubating the developed NMOFs in a concentrated drug aqueous solution (5 mg/ml) at room temperature under constant stirring for 72 h. Afterwards, DOX-loaded NMOFs were pelleted via centrifugation and the supernatant was evaluated for free un-entrapped drug. The results demonstrated a higher EE% as well as LC% for NMOF. The obtained results are in consistency with the previous literature reports which validate that a comprehensive accumulation of the drug within the NMOF structure is hampered by its hydrophilic characteristics^34^. The reduction in loading efficiency of mono-ligated NMOF and dual-ligated NMOF in comparison with the parent NMOF can be explained by the decrease in the available sites for DOX entrance into the porous structure of NMOF due to the NMOF surface shielding by conjugated ligands. This could be ascribed to the steric hinderance imposed by such bulky surface targeting ligands which partially blocking the proper accommodation of relatively large cargo (dimension = 15.3 × 11.9 Å) within the MOF pores (diameter = 18.96 Å). All results are set out in Table 1.

**Table 1:** Indicating both of entrapment efficiency, loading capacity of DOX. HCL to NMOF and FA-NMOF, LA-NMOF, GA-NMOF L-G-NMOF, and cytotoxicity activity of DOX loaded formulations at two-time intervals, (24, 48 h).

Formula	EE %	LC %	IC_50_/24 h ± SD	IC_50_/48 h ± SD
NMOF	50.63 ± 0.96	15.59 ± 0.03	5.982 ± 0.4	2.16 ± 0.16
FA-NMOF	29.77 ± 0.11	9.40 ± 0.05	1.887 ± 0.05	0.838 ± 0.05
LA-NMOF	30.98 ± 0.06	9.84 ± 09	0.641 ± 0.03	0.487 ± 0.07
GA-NMOF	31.63 ± 0.02	9.95 ± 02	0.986 ± 0.08	0.492 ± 0.05
LA-GA-NMOF	19.73 ± 0.71	6.26 ± 04	0.52 ± 0.15	0.32 ± 0.08
Free DOX			1.2 ± 0.25	0.1 ± 0.02

### Zeta potential and in-vitro drug release profiles

**Figure 5:**
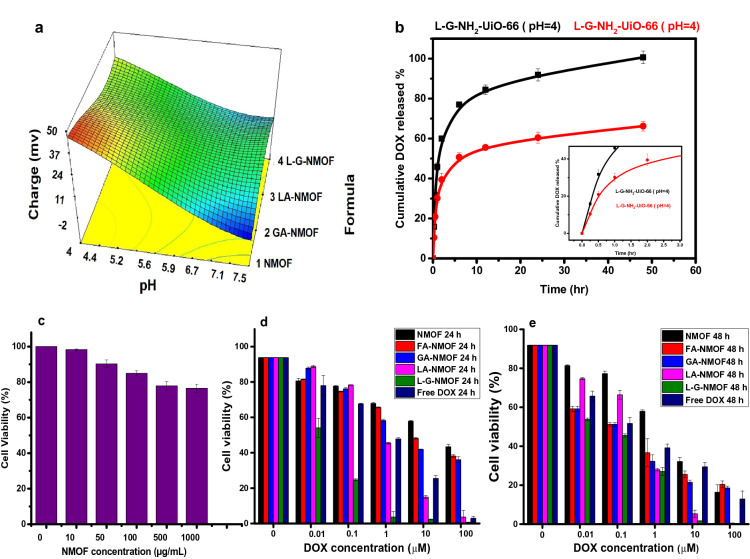
(**a**) chart showing the effect of pH change on the zeta potential values of developed NMOFs formulas, (**b**) In-vitro drug release from LA-GA-NMOF at different pHs (4 and 7.4) with a focus on the release profile during the first 3 h (inset), (**c**) cytocompatibility study using HSF cells post-treatment with different concentrations of pristine NMOF for 72 h. (**d**, **e**) HepG2 cell viabilities post-incubation with free DOX and DOX-loaded NMOFs for 24 h (**d**) and 48 h (**e**).

Measuring surface charge by zeta potential approach is a significant characterization method for NMOF chemical modification due to the considerable changes of charges on their surface after modification. The effect of pH on the NMOF and ligated-NMOF was investigated across a wide range of pH values. Figure 5a demonstrates the effect of change in pH on the surface charge of NMOF and ligated-NMOF. It was apparent that the change in pH has an impact on zeta potential values, where it could be noted that zeta potential decreases by increasing the pH values from acidic to alkaline. NMOF has a high positive charge of 49.7 ± 5.4 at pH 4 and this could be attributed to the protonation of free amino groups on the NMOF surface^27^. Consequently, it is clear that the decrease in the zeta potential values at the same pH in the case of ligated-NMOF was due to consumption of some of the amino groups upon modification with targeting moieties. These results confirm the stability of all formulations in a wide range of pH values as well as the success of immobilization of ligands on the NMOF surface.

As it is commonly known, cancer cells have an acidic pH environment, while normal cells have a neutral pH environment^35^. Guided by the cytotoxicity results, the release behavior from the dual-ligated LA-GA-NMOF nanocarrier was investigated at pH (4 and 7.4) values for mimicking both physiological and tumor cells environment. As shown in Fig. 5b, only 60% of DOX was released after 48 h under neutral pH, compared with nearly 100% of DOX being released under acidic pH over the same time period. The pH-sensitive DOX enhanced release at acidic pH from LA-GA-NMOF might be explained by correlation with the surface zeta potential of drug-loaded NMOF. Zeta-potential measurements showed that all ligated-NMOF exhibited a positive charge at different pH thus, the enhanced DOX release at acidic pH may be attributed to the high positive charge of LA-GA-NMOF at low pH and weak interaction with positively charged DOX. Furthermore, under the acidic pH, the amine groups of DOX become protonated and thereby, increases the solubility of DOX, thus, further increasing the DOX release^36^.

### Biological assessments

#### Biocompatibility and cytotoxicity assessment

Prior to the biological efficacy assessment, the biocompatibility of the DOX-free NMOF was first investigated. Thus, human skin fibroblast (HSF) as a model normal cell-line incubated with NMOF with different concentrations (0, 10, 50, 100, 500 and 1000 µg/ml, respectively) for 72 h was assessed by the SRB assay. As shown in Fig. 5c, it was found that the cells depict high viability ranging from 98 ± 0.42%, and only being reduced to reach 77 ± 0.71% at maximum concentration tested (1000 µg/ml). Thus, all these data prove that the NMOF can be considered as a safe biocompatible carrier for drug delivery.

In this study, DOX.HCl was chosen as a model anticancer drug as it is considered the first line for cancer chemotherapy treatments^37^. In order to evaluate the cytotoxicity of DOX-loaded NMOFs, HepG2 cell were incubated with different formulations for two different time intervals; 24 h and 48 h. The obtained results exhibited an increasing antitumor activity upon the using of different active targeting moieties. As shown in Fig. 5d and e, all DOX-loaded formulations exhibited a time-dependent toxicity which was reflected by the reduction of IC_50_ values post-48 h as compared to 24 h. Further, the obtained results confirmed that the DOX-loaded mono-ligated NMOF exhibited significantly lower IC_50_ values in comparison to the non-ligated NMOF at both time intervals with the following order LA-NMOF displayed the lowest IC_50_ amongst the tested monoligated NMOF followed by GA-NMOF and finally FA-NMOF (*p* ≤ 0.001). These findings demonstrated that the targeting moieties decorating the NMOF surface could selectively enhance antitumor activity against HepG2 cells. Based on the order of activity of the mono-ligated NMOFs, the LA-GA dual ligated (LA-GA-NMOF) was prepared and evaluated, and it attained a significantly low IC_50_ (0.52 ± 0.15, 0.32 ± 0.08, *p* ≤ 0.001) at both time intervals (24 and 48 h) as compared to FA-NMOF and the non-ligated NMOF.

Moreover, in comparison to the free DOX.HCl, the dual ligated NMOF had a significantly higher cytotoxicity at 24 h (IC50 = 1.2 ± 0.25 µM, *p* ≤ 0.001). Additionally, after 48 h of treatment the dual ligated NMOF proved to be the closest to the free drug cytotoxicity (0.1 ± 0.02 µM). The superiority of the dual-ligated systems compared to the mono-ligated systems could be ascribed to their enhanced cellular uptake, by interaction of the ligands to the corresponding receptors on HepG2 cells (GA receptors and asialoglycoprotein receptors which are over-expressed in HepG2 cells). All cytotoxicity data mentioned in Table 1.

#### Cellular uptake results

**Figure 6:**
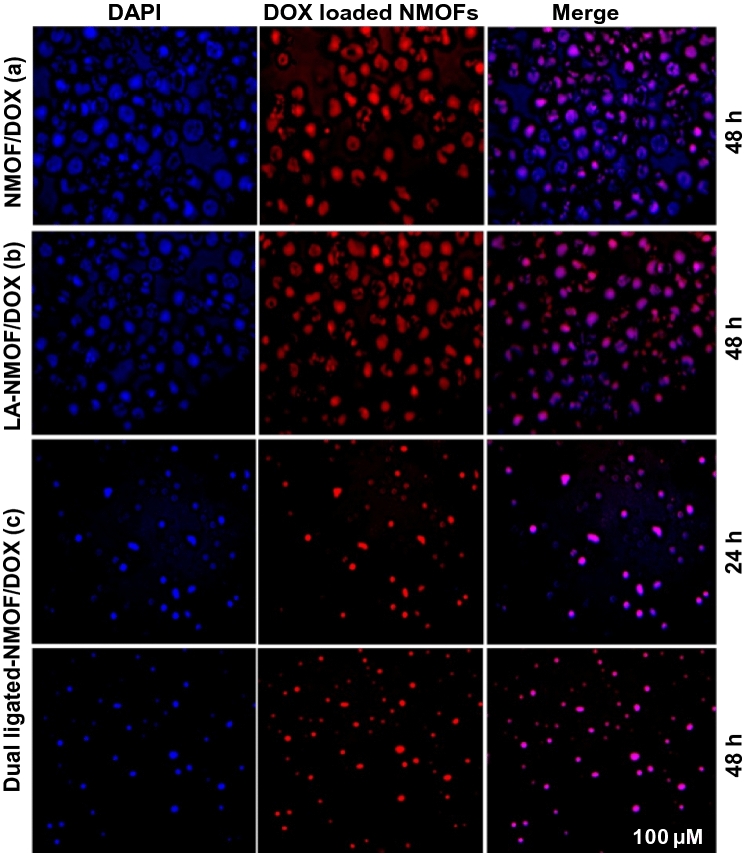
Fluorescence micrographs of HepG2 cell lines treated with (**a**) NMOF-DOX at 48 h. (**b**) LA-NMOF-DOX at 48 h, (**c**) LA-GA-NMOF-DOX at 24 h and 48 h. Scale bar 100 µM and magnification 10 ×.

The cellular uptake and localization of the NMOF, LA-NMOF, LA-GA dual-ligated NMOF were evaluated using fluorescence microscopy, being tested on HepG2 cells at two-time intervals; 24 h and 48 h as indicated in Fig. 6. The results showed red fluorescence confirming the efficient cellular uptake and internalization of the DOX-loaded formulations. The nuclear localization of the evaluated formulations was confirmed in the merged pictures of stained cells with DAPI and DOX. As shown in the figure, the nuclear localization of ligated NMOF was enhanced in comparison to the non-ligated NMOF. Also, Fig. 6c results showed an enhancement and time-dependent cellular uptake of DOX by dual-ligated NMOF. These findings were in agreement with the obtained cytotoxicity results.

#### Flow cytometric analyses

**Figure 7:**
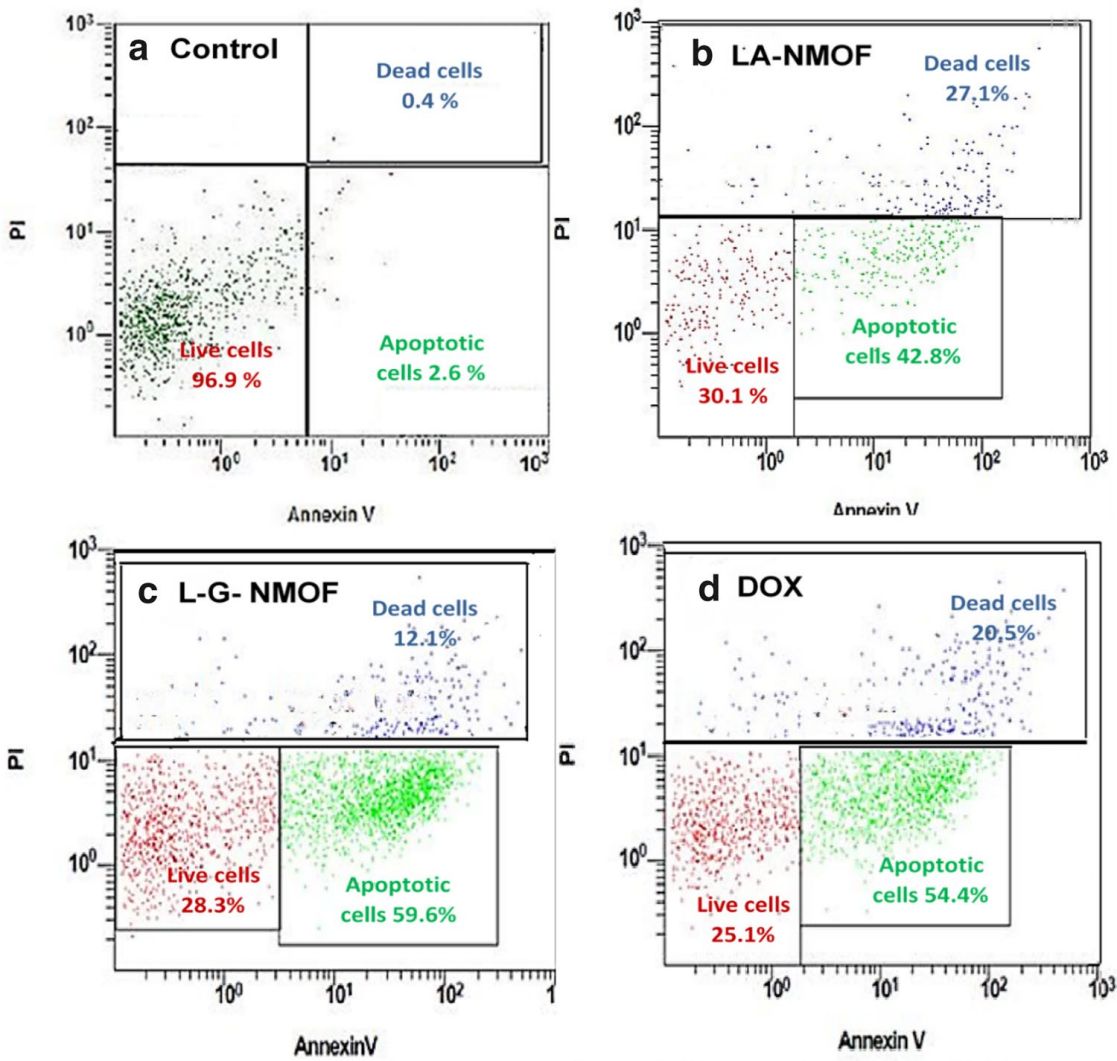
Flow cytometric histograms of (**a**) untreated HepG2 cell lines, (**b**) HepG2 cell lines treated with LA-NMOF-DOX, (**c**) HepG2 cell lines treated with LA-GA-NMOF-DOX, and (**d**) HepG2 cell lines treated with free DOX.

The ability of the ligated NMOFs to induce apoptosis was studied using flow cytometry. Treated HepG2 cells are classified into three different populations the first one represents early apoptotic cells which are strongly stained by annexin-V fluorescent dye due to the abundant phosphatidyl choline binding. Secondly, dead cells population which possess poor membrane integrity thus extensively stained by propidium iodide (PI) including both late apoptotic as well as necrotic cells. Finally, the last third cell population represents the alive cells which are negative for both annexin V and PI. As shown in Fig. 7, compared to the non-treated control group, the results showed that treatment of HepG2 cells with DOX-loaded NMOF stimulated a cell death in cells with a significance in cell reduction for both of mono-ligated NMOF (30.1 ± 2.4%) and dual-ligated NMOF (29 ± 1.99) with (*p* < 0.001). Moreover, the dual-ligated NMOF showed higher significant (59.4 ± 1.2) apoptotic effect compared with mono-ligated NMOFs (42.4 ± 0.98%,) and the untreated control (2.6 ± 0.68%, *p* < 0.001). At the meantime, the apoptotic cell death caused by the dual-ligated NMOF was higher than that of DOX treatment (positive control group, 54.2 ± 1.31%, *p* < 0.001), proving the efficacy of the developed dual-ligation in killing hepatic cancerous cells. Additionally, dual-ligated NMOF showed higher significant necrotic effect (12.2 ± 1.99%) which is 30.25-fold more than their untreated control (0.4 ± 0.01, *p* < 0.001). The findings of this study further prove the superiority of the developed dual-ligated NMOF as a delivery system for hepatic cancer treatment.

## Conclusion

In conclusion, the present study involved the design and development of a hepatic tumor-targeting agent-bearing NMOF drug delivery systems (DDSs) as multifunctional systems. The targeting moieties were covalently decorated to Zr-based NMOF either as mono-ligated or dual-ligated via EDC/NHS reaction. The success of targeting molecules conjugation was experimentally confirmed, and the biocompatibility of the NMOFs was verified on normal cells. In-vitro release of DOX from MOFs showed pH-dependency, in which the release profile was faster at an acidic environment that mimics the tumor environment. Furthermore, cell imaging and flowcytometry results clarified that the DOX-loaded LA-GA-NMOF exhibited the best anticancer activity compared to DOX-loaded NMOF and all DOX-loaded monoligated NMOFs. Conclusively, this work demonstrates that the newly-developed dual-ligated LA-GA-NMOF has a potential promise for selective targeting and efficient drug delivery to treat liver localized tumors.

## Supplementary Information

Supplementary Information.
