# Supplementary material for: Dual-ligated metal organic framework as novel multifunctional nanovehicle for targeted drug delivery for hepatic cancer treatment
Source: Sci Rep. 2021 Oct 6;11:19808. doi: 10.1038/s41598-021-99407-5 (PMC8494812; doi:10.1038/s41598-021-99407-5)
Supplement: Supplementary file 1 — Supplementary Information. [file 41598_2021_99407_MOESM1_ESM.docx]

**Dual-ligated Metal Organic Framework as Novel Multifunctional Nanovehicle for Targeted Drug Delivery for Hepatic Cancer Treatment**

Mostafa Fytory, ^1,2^, Kholoud K. Arafa^1^, Waleed El Rouby ^2^, AA Farghali ^2^, Mahmoud Abdel-Hafiez^3^ and Ibrahim M. El-Sherbiny ^1, *^

*^1^Nanomedicine Labs, Center for Materials Science (CMS), Zewail City of Science and Technology, Giza 12578, Egypt; ^2^ Material Science and Nanotechnology Department, Faculty of Postgraduate Studies for Advanced Sciences (PSAS), Beni-Suef University, 62511 Beni-Suef, Egypt* ^3^ Department of Physics and Astronomy, Uppsala University, Box 516, SE-75120 Uppsala, Sweden.

**^*^Corresponding author:**

Prof. Dr. Ibrahim M. El-Sherbiny

Email: [ielsherbiny@zewailcity.edu.eg](mailto:ielsherbiny@zewailcity.edu.eg)

**Table S1.** Chemical synthesis molar ratios for preparation of different plain and ligated NMOFs formulations.

| **Molar Ratio** | **NMOF** | | **FA** | | **LA** | | **GA** | | **EDC.HCL** | | **NHS** | | |  |
| --- | --- | --- | --- | --- | --- | --- | --- | --- | --- | --- | --- | --- | --- | --- |
|  | **mg** | **mmol** | **mg** | **mmol** | **mg** | **mmol** | **mg** | **mmol** | **mg** | **mmol** | | **mg** | **mmol** | |
| **1/2** | 100 | 0.084 | 18 | 0.042 | 15.21 | 0.042 | 19.78 | 0.042 | 20.12 | 0.105 | | 10.1 | 0.087 | |
| **1/4** |  |  | 9 | 0.021 | 7.52 | 0.021 | 9.88 | 0.021 | 10.06 | 0.05 | | 5.1 | 0.43 | |
| **1/8** |  |  | 4.5 | 0.011 | 3.76 | 0.011 | 4.94 | 0.011 | 5.03 | 0.025 | | 2.5 | 2.5 | |
